# Senescent cells enhance ischemic aging in the female heart

**DOI:** 10.18632/aging.204585

**Published:** 2023-03-23

**Authors:** Daniele Torella, Nadia Salerno, Eleonora Cianflone

**Affiliations:** 1 Department of Experimental and Clinical Medicine, Magna Graecia University, Catanzaro 88100, Italy; 2 Department of Medical and Surgical Sciences, Magna Graecia University, Catanzaro 88100, Italy

**Keywords:** cell senescence, aging, cardiac stem cells, cardiac regeneration, female sex

Aging negatively affects tissue cellular homeostasis and regeneration, in part due to resident stem cell-intrinsic accumulation of damage and dysfunction, similar functional deterioration of niche cells (tissue-specific cell types, microvascular, fibroblasts and immune cells) and increased inflammation (inflammageing) of the microenvironment [1].

Cellular senescence, a key feature of aging, is classically reported as a passive cell state whereby senescent cells irreversibly cease proliferation, while it is instead a more dynamic and active cell state as senescent cells produce and secrete soluble factors (the so-called, senescence-associated secretory phenotype, SASP) that can influence neighboring cells and tissues [1]. Senescent cells make up a small percentage of tissue cells, even in older individuals, and yet they cause major damage by secreting these signaling proteins through the SASP. The SASP induces inflammation and fibrosis, and it hampers the functions of healthy neighboring cells, reducing overall tissue regeneration. Because of the SASP, senescent cells are thought to contribute to many diseases and unwanted side effects of ageing.

Clinical and experimental evidence have associated cellular senescence, senescent cell accumulation, and the production and release of SASP components with age-related cardiovascular disease (CVD) such as myocardial ischaemia and infarction, diabetic cardiomyopathy and cancer drug-related cardiotoxicity and resulting heart failure [1–4]. However, the precise role of senescent cells in these conditions is unclear and, in some instances, both detrimental and beneficial effects have been reported [1–4]. Senescent cell transient appearance in the heart in response to temporary stress can be beneficial, and acute cellular senescence has important physiological roles in heart development and regeneration; however, as senescent cells accumulate progressively in the heart during ageing they cause an age-related decline in heart function [1, 2].

Gender (chromosomal sex) in mammals is a risk factor in many conditions, including aging, cardiovascular diseases, neurodegenerative diseases, cancer, and more. For each of these, males and females display distinct traits in disease development and progression. To date, studies on the cellular and molecular basis of such differences have largely focused on the role of sex hormones [5]. Intriguingly, new research suggests that cellular senescence may underlie sex differences in several pathologies [5]. If cellular senescence is a driver of pathophysiological changes in various body tissues, the question is whether cellular senescence is differently regulated in males vs females and therefore whether a sex-dependent accumulation of senescent cells is responsible for the sex differences in clinical phenotypes. To date, it has been demonstrated that female sex is associated with a lower capacity for DNA repair throughout the lifespan [5]. Furthermore, it has been shown the existence of sex differences in senescence onset whereby female cells have a greater tendency to undergo cellular senescence in response to genotoxic stress [5]. On the other hand, senescence cell number is greater in male mice until the end of life. Sex steroid hormones regulate both senescence-dependent DNA damage and senescence regulatory pathways [5]. Overall, these findings suggest that biological sex may differently regulate cellular senescence, but this hypothesis remains to be proven.

Important sex-specific differences in aging and the aged phenotype exist but the mechanism(s) by which this sex-related age interaction influences CVD development and progression remains elusive. Women live longer than men but there is a paradox. Women are frailer and have worse health at the end of life than men whereby older females are reported to be at a greater risk for CVD than age-matched men.

**Figure 1:**
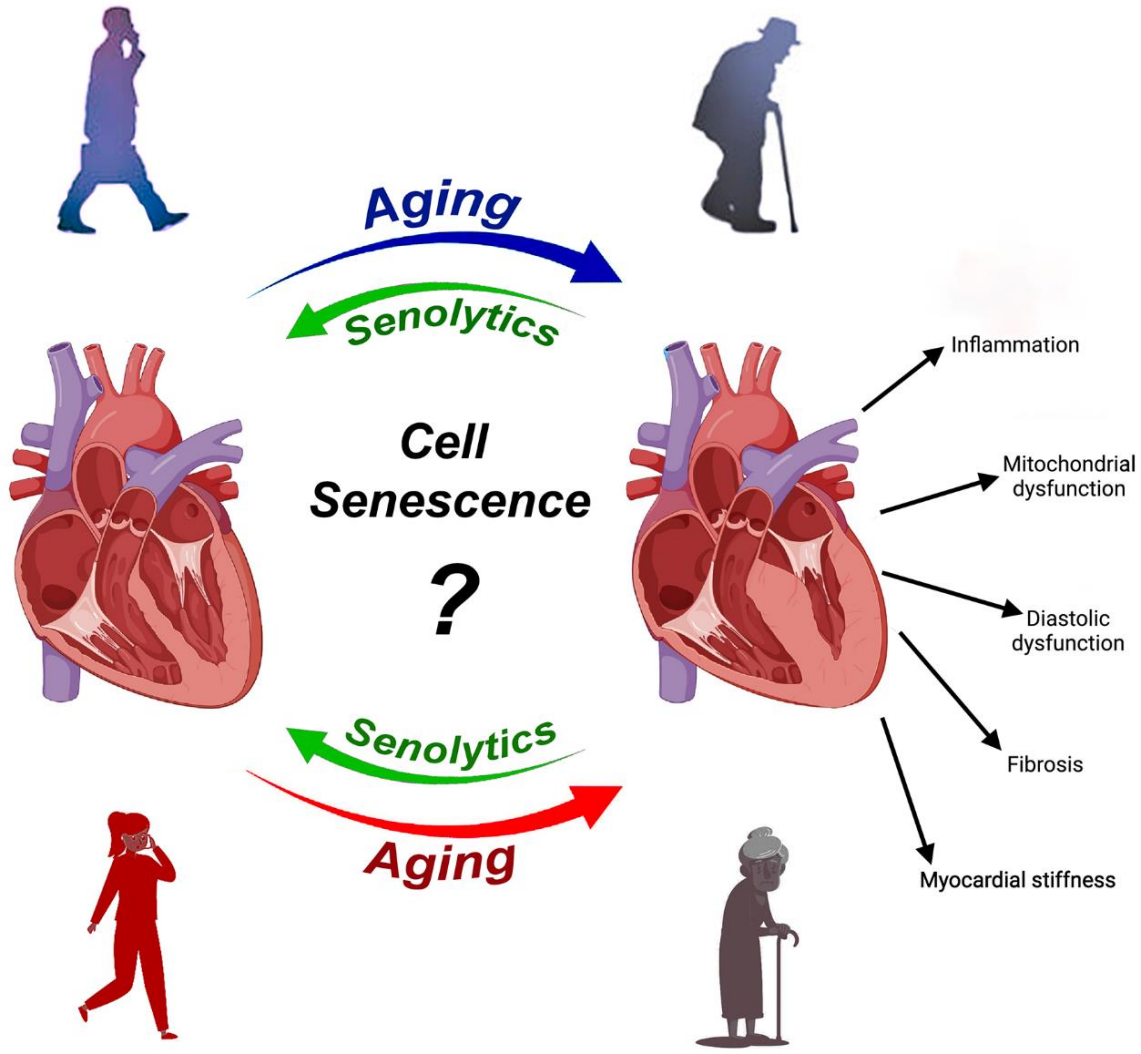
**Aging results in structural deteriorations and functional decline of the heart**. One central mechanism of aging is cell senescence and several studies have provided evidence that clearing senescent cells with senolytics in male rodents ameliorate cardiac decline and dysfunction with aging and in aged mice with ischemic cardiomyopathy. That senescence is central to cardiac aging in female sex remains unaddressed. Nevertheless, we demonstrated for the first time that senescence clearance by senolytics improves cardiac remodeling and function after myocardial infarction in very old female mice [7].

Recently, it has been demonstrated that compounds termed senolytics, due to their ability to preferentially induce apoptosis in senescent cells, eliminate senescent cells preventing or reversing age-related CVD [6]. In particular, senolytics have an effective pro-apoptotic potential by targeting members of the Bcl-2 family, p53/p21Cip, ephrins, the phosphatidylinositol-4,5-bisphosphate 3-kinase (PI3K), plasminogen -activated inhibitor-1 and 2 (PAI1 and 2) and hypoxia-inducible factor-1α [6]. Administration of senolytics to aged (24-month-old) male mice reduces SASP components and cardiomyocyte (CM) senescence. It attenuates all characteristics of pathological ventricular remodeling reducing CM hypertrophy, interstitial fibrosis and left ventricle (LV) mass [6]. Senolytics treatment results in a significant improvement in ejection fraction (EF) [6], suggesting that the clearance of senescent cells underlies the observed benefits in males. Unfortunately, however, no specific data exists for the effects of senolytics on senescent cells removal and cardiac remodeling and function in females with CVD. For this reason, it is still unknown whether cell senescence and its associated SASPs also plays a significant causal role in the female aged heart (Figure 1).

To close this gap, in a recent study from our group, we administered the combination of two senolytics, dasatinib and quercetin (D+Q) or just their vehicle to 22-24 months old C57BL/6 female mice after myocardial infarction (MI) [7]. This senolytics combination has been proven effective in counteracting age effects on male mouse hearts but also on other tissues like the skeletal muscle [6, 8]. We have shown that female cardiac aging is characterized by accumulation of cardiac senescent cells that are further increased by MI. Accordingly, D+Q improved global LV function and myocardial performance in these aged female mice after MI. Despite their terminally differentiated state, cardiomyocytes also acquire a senescent phenotype in aged females. D+Q removed senescent cardiac myocyte and non-myocyte cells, ameliorating cardiac remodeling and regeneration in the aged female mice. Furthermore, senolytics removed aged-dysfunctional cardiac stem/progenitor cells (CSCs), relieving healthy CSCs with normal proliferative and cardiomyogenic differentiation potential. In conclusion, our data show that, like reported for males, cardiac senescent cells accumulate in the aged female hearts and that removing senescent cells is a key therapeutic target for efficient repair of the aged female heart [7].

These data fill a gap of knowledge in the role of biological sex in determining senescence and cardiac muscle dysfunction with age, providing robust evidence that indeed female cardiac aging is driven by senescence. Concurrently, accumulating senescent cells are detrimental for the old female heart after ischemic injury as senescent cells removal by senolytics ameliorates cardiac remodeling after MI improving heart function. On the other hand, these data go against the view that senescence is at least in part a positive response to limit fibroblast proliferation limiting reactive fibrosis after MI.

Nevertheless, relevant questions for the aging biology of the heart but also for all other body tissues remain to be addressed. Is senescence an irreversible phenotype or a functional and reversible one? What is the role of the chromosomal sex vs the hormonal milieu? Are these two views mutually exclusive and how are they regulated by biological sex? Is senescence mainly a response to damage or an irreversible cell fate independent from injury (despite being increased by injury)? Is therefore senescence stochastic or programmed? Are senescence cells heterogenous and if so, what this heterogeneity means? Is it senescence of tissue-specific stem cells or is it senescence of niche cells in the tissue microenvironment to dictate regenerative defect of the relative aged organ? More importantly, does “reversing” the aged phenotype with senolytics increases lifespan of either sex? Clearly, answering these questions will pave the way to a coherent and informed translation of senolytics therapy to first-in-human trial on aging and CVD. Lastly, the sex-related differences in the aged phenotype emphasize the need to include both sexes in the experimental design to address the questions raised above.
